# The Burden of Acute Febrile Illness Attributable to Dengue Virus Infection in Sri Lanka: A Single-Center 2-Year Prospective Cohort Study (2016–2019)

**DOI:** 10.4269/ajtmh.21-0604

**Published:** 2021-11-01

**Authors:** Hasitha Tissera, Preshila Samaraweera, Melanie de Boer, Sanjay Gandhi, Ludovic Malvaux, Shailesh Mehta, Paba Palihawadana, Valerie Vantomme, Robert Paris, Alexander Schmidt

**Affiliations:** ^1^ Epidemiology Unit, Ministry of Health, Colombo, Sri Lanka;; ^2^ National Dengue Control Unit, Ministry of Health, Colombo, Sri Lanka;; ^3^ GSK, Rockville, Maryland;; ^4^ GSK, Mumbai, India;; ^5^ GSK, Rixensart, Belgium;; ^6^ GSK, Wavre, Belgium

## Abstract

We performed a 2-year prospective cohort study to determine the incidence of dengue in Angoda, Colombo district, Sri Lanka (NCT02570152). The primary objective was to determine the incidence of acute febrile illness (AFI) because of laboratory confirmed dengue (LCD). Secondary objectives were to determine AFI incidence because of non-LCD, describe AFI symptoms, and estimate AFI incidence because of LCD by dengue virus (DENV)-type and age group. Participants from households with at least one minor and one adult (≤50 years) were enrolled and followed with scheduled weekly visits and, in case of AFI, unscheduled visits. Blood was collected for DENV detection at AFI visits, and symptoms recorded during the 7-day period following AFI onset. A total of 2,004 participants were enrolled (971 children, and 1,033 adults). A total of 55 LCD episodes were detected (overall incidence of 14.2 per 1,000 person-years). Incidence was the highest among children < 5 years (21.3 per 1,000 person-years) and 5–11 years (22.7 per 1,000 person-years), compared with adults ≥ 18 years (9.2 per 1,000 person-years). LCD was mostly (83.6%) caused by DENV-2 (*n* = 46), followed by DENV-1 (*n* = 6) and DENV-3 (*n* = 3). Common symptoms of LCD were headache, fatigue, myalgia, loss of appetite, and arthralgia. Incidence of AFI because of non-LCD was 47.3 per 1,000 person-years. In conclusion, this study reports the LCD incidence for a DENV-2 dominated epidemic that is comparable to the incidence of suspected dengue reported passively for 2017, one of the worst outbreaks in recent history.

## INTRODUCTION

Dengue is an arboviral illness caused by one of four different serotypes of infecting virus (dengue virus [DENV]-1, DENV-2, DENV-3, and DENV-4). The main vector is *Aedes aegypti*, a mosquito that is highly adapted to urban environments. Symptoms of DENV infection include fever, headache, skin erythema, arthralgia, and myalgia, which are also common symptoms of other febrile illnesses; thus, misdiagnosis and underreporting are common. Dengue has three phases: a febrile phase, a critical phase, and a recovery phase, of which the febrile phase has a sudden onset and lasts about 2–7 days. The clinical manifestations have a wide range, from mild fever to more severe dengue hemorrhagic fever and potentially fatal dengue shock syndrome.^1^

Dengue is the most important mosquito-borne viral disease globally, of which the incidence has increased 30-fold during the past five decades.^1^ Geographically, half the world’s population is at risk of DENV infection, and it was estimated that in 2013, globally, 58.4 (uncertainty interval 23.6–121.9) million people were infected.^2^ Although the disease’s exact distribution is uncertain, dengue is found in tropical and subtropical climates, mostly in urban and semiurban areas.^3^ According to the World Health Organization (WHO), an estimated 100 countries are affected,^4^ although there is strong evidence for dengue occurrence in 128 countries.^5^ About 70.0% of the disease burden is in Asia.^6^

According to the WHO *Global Strategy for Dengue Prevention and Control*, improved surveillance is needed to estimate dengue disease burden better.^4^ Improved surveillance would benefit reporting, prevention, and control of dengue, whereas incidence rates and clinical data would also support the definition of clinical endpoints in future vaccine efficacy trials.

This prospective cohort study was designed to support the selection and definition of clinical and laboratory endpoints for future dengue vaccine efficacy trials and provide incidence data for sample size calculation in Sri Lanka. The study’s primary objective was to determine the incidence of acute febrile illness (AFI) because of laboratory confirmed dengue (LCD) in the study population. Secondary objectives were to determine the incidence of AFI because of non-LCD, describe the signs and symptoms of AFI, and estimate the incidence of AFI because of LCD by DENV-type and age group.

## METHODS

### Study design.

**Figure 1.**
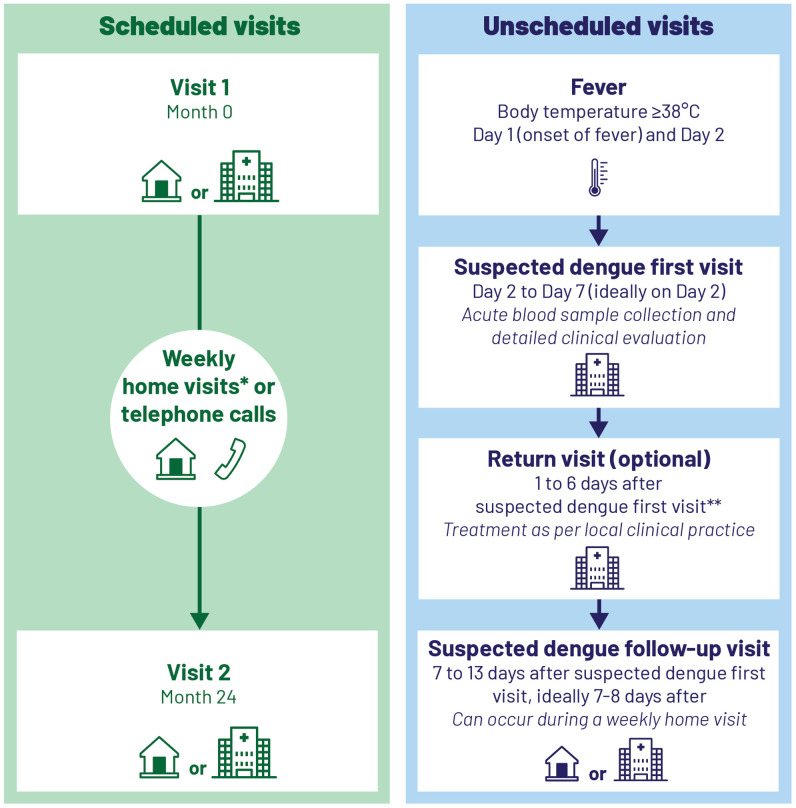
Study design. * At least every other week. ** As per investigator judgment. This figure appears in color at www.ajtmh.org.

This study was a prospective, community-based, cohort study performed in an urban Kolonnawa Medical Officer of Health (MOH) division in the Colombo district, Sri Lanka, by household sampling. The study period was from June 1, 2016 to June 28, 2019. Recruitment of the participants took place from June 1, 2016 to June 28, 2017 after which each participant was followed for 2 years. Follow-up of the last patient was completed on June 28, 2019. The study consisted of scheduled clinic visits, including the enrollment visit (Visit 1) and the close-out visit (Visit 2), and weekly contacts. The weekly contacts consisted of home visits at least every other week and telephone contacts. In addition, in the case of AFI, an unscheduled Suspected Dengue Visit, optional Return Visit, and Follow-up Visit were also performed. AFI was defined as body temperature ≥ 38°C on ≥ 2 consecutive days, measured at least twice, and at least 8 hours apart. Blood samples were only collected during the Suspected Dengue Visit. The study design is illustrated in Figure 1.

In the event of AFI, blood samples were collected for participants ≥ 5 years of age. For participants < 5 years of age, samples were collected only if clinically indicated, as per investigator judgment. Depending on the patient’s age, pediatric or adult sample collection kits were provided that included vials for dengue nonstructural protein 1 (NS1)-rapid antigen test, confirmatory reverse transcriptase-polymerase chain reaction (RT-PCR), and complete blood count. A total of 5 mL blood was collected into individual vials using the sampling kit identified with a unique number for each case and episode. A designated laboratory technician under the supervision of a specialist microbiologist/hematologist performed both NS1-rapid antigen test and complete blood count at the infectious disease hospital laboratory on the same day, while the serum samples for RT-PCR were stored at −80°C at the hospital laboratory. Batches of samples were shipped monthly to a designated laboratory in the United Kingdom for RT-PCR testing and dengue virus serotyping. LCD was defined as AFI with laboratory confirmation of dengue by RT-PCR using the Simplexa Dengue kit (Focus Diagnostics, San Juan Capistrano, CA; Simplexa is a trademark owned by or licensed to Focus Diagnostics) on the acute serum sample taken during the 7-day period (Days 2–7) from the onset of fever.

The study was conducted following the International Council for Harmonization of Good Clinical Practice guidelines^7^ and the Declaration of Helsinki. The study protocol, amendments, and other study-related documents were reviewed and approved by the Ethics Review Committee of the Faculty of Medicine at the University of Colombo, Sri Lanka. The study was registered at clinicaltrials.gov (NCT02570152).^8^

### Participants.

The study population included participants living in randomly selected households in geographically defined communities at Kolonnawa, Colombo, Sri Lanka. In the Kolonnawa MOH division, six areas were selected randomly from a total of 46 smallest administrative areas. A list of randomly selected households was prepared according to probability-proportional-to-size sampling of the population using the census register in each of the six areas. Trained research assistants approached the households. Selected households were considered eligible for inclusion if at least one household member ≥ 6 months to < 18 years of age assented and at least one adult (≤ 50 years) who consented for himself/herself and the minor in the household to participate in the study. The protocol targeted 2,000 participants to be enrolled.

The inclusion criteria involved 1) in the opinion of the investigator, the participant (or participant’s parent[s]/legally acceptable representative [LAR]) and the minor were able to participate and would comply with the requirements of the protocol; 2) signed/thumb-printed informed consent (and assent if applicable) was obtained from the participant/participant’s parent(s)/LAR(s); 3) the participant was part of a household with at least one child (≥ 6 months and < 18 years of age) and at least one adult (≤ 50 years of age) of which informed consent (and assent if applicable) was obtained; and 4) participants planned—at the time of enrollment—to remain in the same household during the study period.

The exclusion criteria involved: 1) child in care; 2) participation in another epidemiologic study or clinical trial that would conflict with the current study, based on the investigator’s judgment; and 3) terminal illness or mental incapacity based on the investigator’s judgment.

### Statistical analysis.

Descriptive statistics (mean, standard deviation [SD], and frequency) were calculated for sociodemographic characteristics. There was a single analysis set (total cohort) used in this study that included all participants enrolled. The incidence rate of first AFI because of LCD was calculated along with the 95% confidence interval (CI), based on the total cohort. The incidence rate of first AFI because of LCD by serotype (DENV-1 to -4) and incidence rate of all AFI because of non-LCD with 95% exact CI were calculated for overall and age group, based on the total cohort.

A description of signs and symptoms of AFI because of LCD and because of non-LCD included the percentage of AFI presenting each sign or symptom (any intensity and grade 3) during the 7-day period from the onset of AFI. Duration of signs and symptoms were also described for LCD and non-LCD episodes. Analyses were performed, overall and separately for each age group. Univariate and multivariate stepwise logistic regressions were performed to investigate the association of signs and symptoms with AFI. Grading of symptoms is specified in the Supplemental Material (Supplemental Table 1).

We assumed a drop-out rate of 10.0% in the first year and a drop-out rate of 20.0% in the second year for the sample size calculation. If a maximum of 2,000 participants were enrolled for a 2-year follow-up period, the final analysis would be performed on approximately 3,300 person-years overall. Participants withdrawn or lost to follow-up were not replaced, and missing data were not imputed. Age was determined at the time of enrollment. The statistical analyses were performed using the Statistical Analysis System Version 9.4 (SAS Institute Inc., Cary, NC).

## RESULTS

### Baseline characteristics.

A total of 2,004 participants (total cohort) were enrolled. About half of the participants were adults 18−50 years of age (*n* = 1,033; 51.6%), the mean age was 22.3 years (SD ± 15.1), and about half were female (*n* = 1,038; 51.8%). The majority of the participants had a secondary school education (*n* = 1,368; 68.3%) and a medium social economic status (*n* = 1,853; 92.5%) (Table 1). Of the 2,004 participants who enrolled, 1,989 (99.3%) completed the 2-year follow-up study. A total of 15 participants were withdrawn: two participants because of noncompliance with protocol requirements (one participant was unavailable for study visits from June 14, 2018, and one participant could not be reached as of December 12, 2018); and 13 participants because they moved from the study area (Table 1). There was a total number of 3,869.3 person-years of follow-up for the 2,004 participants (Table 2).

**Table 1:** Demographic and households’ characteristics (total cohort)

Demographic characteristic	Participants
	*N* = 2,004
Age at enrollment, mean ± SD in years	22.3 ± 15.1
Median in years (range)	19.0 (6 months–49 years)
Age groups, *n* (%)	
<5 years of age	257 (12.8)
5–11 years of age	433 (21.6)
12–17 years of age	281 (14.0)
≥18 years of age	1,033 (51.6)
Female gender, *n* (%)	1,038 (51.8)
Education, *n* (%)	
None	2 (0.1)
Primary school	359 (17.9)
Secondary school	1,368 (68.3)
Graduate	24 (1.2)
Postgraduate	7 (0.3)
Not applicable (for participants < 5 years of age)	243 (12.1)
Missing	1 (0.05)
Social Economic Status, *n* (%)	
Low	136 (6.8)
Medium	1,853 (92.5)
High	15 (0.7)
Withdrawn participants, *n* (%)	
Noncompliance with protocol requirements	2 (0.1)
Moved from the study area	13 (0.6)

*N* = total number of participants; *n* = number of participants in a given category.

**Table 2:** Incidence rate of AFI because of LCD or non-LCD overall and by age group

			AFI because of LCD			AFI because of non-LCD		AFI because of undetermined cause
Age group	*N*	Person-years	*n*	Incidence rate per 1,000 person-years (95% CI: LL–UL)	*n*	Incidence rate per 1,000 person-years (95% CI: LL–UL)	*n*	Incidence rate per 1,000 person-years (95% CI: LL–UL)
< 5 years of age	257	517.66	11	21.25 (10.61–38.02)	54	104.31 (78.36–136.11)	3	5.80 (1.20–16.94)
5 to 11 years of age	433	837.25	19	22.69 (13.66–35.44)	71	84.8 (66.23–106.97)	5	5.97 (1.94–13.94)
12 to 17 years of age	281	548.47	7	12.76 (5.13–26.30)	24	43.76 (28.04–65.11)	3	5.47 (1.13–15.98)
≥ 18 years of age	1,033	1,965.88	18	9.16 (5.43–14.47)	34	17.3 (11.98–24.17)	2	1.02 (0.12–3.68)
Overall	2,004	3,869.26	55*	14.21 (10.71–18.50)	183	47.3 (40.69–54.67)	13	3.36 (1.79–5.75)

AFI = acute febrile illness; Incidence rate per 1,000 person-years = number of first event per 1,000 person-years; LCD = laboratory confirmed dengue; LL = lower limit; *N* = number of participants at risk during the follow-up period; *n* = number of first events reported during the follow-up period at risk; Person-years = the sum of the years of observation of all participants within the age category expressed in years, censored at the first event; UL = upper limit.

* Of which two episodes were reported in one individual.

### Most AFI episodes were because of non-LCD.

During the 2-year follow-up, 251 AFI episodes were reported. Based on the RT-PCR test, 55 of these were because of LCD, 183 were because of non-LCD (Table 2), and 13 episodes were undetermined. Of the 13 episodes with undetermined results, three occurred in participants < 5 years of age, five in participants 5–11 years of age, three in participants 12–17 years of age, and two in participants ≥ 18 years of age (Table 2). The AFI cause remained undetermined when no validated PCR results were available.

### Incidence rate of dengue (AFI because of LCD).

A total of 55 AFI episodes as a result of LCD were reported over the 2-year study period, of which two episodes were reported in one individual. The incidence rate of AFI because of LCD was 14.2 per 1,000 person-years (95% CI: 10.71–18.50) in the total cohort (Table 2). The incidence rate was the highest in children 5–11 years of age at 22.7 per 1,000 person-years (95% CI: 13.66–35.44) and children < 5 years of age at 21.3 per 1,000 person-years (95% CI: 10.61–38.02), while it was the lowest in adults ≥ 18 years of age at 9.2 per 1,000 person-years (95% CI: 5.42–14.47) (Table 2).

### Incidence rate of AFI because of non-LCD.

A total of 183 AFI episodes as a result of non-LCD were reported over the 2-year study period. No additional testing was performed to identify the cause of AFI in the non-LCD cases. The incidence rate of AFI because of non-LCD was 47.3 per 1,000 person-years (95% CI: 40.69–54.67) in the total cohort. The incidence rate was highest in children < 5 years of age at 104.3 per 1,000 person-years (95% CI: 78.36–136.11) and lowest in adults at 17.3 per 1,000 person-years (95% CI: 11.98–24.17) (Table 2).

### Most LCD episodes were caused by DENV-2.

Of the 55 LCD episodes, 83.6% (*n* = 46) were caused by DENV-2, 10.9% (*n* = 6) were because of DENV-1, and 5.5% (*n* = 3) were because of DENV-3. The DENV-4 episodes were not reported. The incidence rate per 1,000 person-years was 1.6 for DENV-1 (95% CI: 0.57–3.38), 11.9 for DENV-2 (95% CI: 8.71–15.86), 0.8 for DENV-3 (95% CI: 0.16–2.27), and 0.0 for DENV-4 (95% CI: 0.00–0.95) (Supplemental Table 2).

### Symptoms of AFI because of LCD.

During the 7-day follow-up period after the onset of an AFI because of LCD, the most commonly reported symptoms of interest (i.e., those occurring in at least 70.0% of the AFI episodes) included headache (over 90.0% of episodes on any given day), fatigue, myalgia, loss of appetite, and arthralgia (Table 3). The incidence of each of these symptoms remained quite stable throughout the 7-day follow-up, with similar incidences from Day 1 through Day 7. Bleeding was reported in less than 8.0% of episodes (4/55 episodes) (Table 3).

**Table 3:** Symptoms of any intensity with AFI because of LCD during the 7-day period from the onset of fever

	Day 1–5		Day 6–7	
Symptoms	*n*	%	*n*	%
Headache/irritability	51	92.73	50	90.91
Fatigue/decrease in everyday activity	48	87.27	48	87.27
Myalgia (muscle pain)	47	85.45	47	85.45
Loss of appetite	43	78.18	43	78.18
Arthralgia (joint pain)	42	76.36	42	76.36
Eye pain	34	61.82	34	61.82
Reduced fluid intake	33	60.00	33	60.00
Abdominal pain	31	56.36	31	56.36
Nausea	29	52.73	29	52.73
Vomiting	29	52.73	29	52.73
Rash	5	9.09	5	9.09
Any bleeding (skin, mouth, anus)	4	7.27	4	7.27

AFI = acute febrile illness; LCD = laboratory confirmed dengue; *n* = number of AFI episodes presenting with a sign or symptom of any intensity in a given category; % = *n*/55 × 100.

### Symptoms of grade 3 intensity of AFI because of LCD.

The most common symptoms with grade 3 intensity during the 7-day follow-up period, which prevented normal daily activities such as attending school or work were fatigue (25.5%), headache/irritability (20.0%), myalgia (18.2%), arthralgia (14.6%), and loss of appetite (14.6%). The incidence of each of the grade 3 signs and symptoms remained stable throughout the 7-day follow-up (Table 4).

**Table 4:** Symptoms of grade 3 intensity with AFI because of LCD during the 7-day period from the onset of fever

	Day 1–7	
Symptoms	*n*	%
Fatigue/decrease in everyday activity	14	25.45
Headache/irritability	11	20.00
Myalgia (muscle pain)	10	18.18
Arthralgia (joint pain)	8	14.55
Loss of appetite	8	14.55
Reduced fluid intake	4	7.27
Eye pain	2	3.64
Abdominal pain	2	3.64
Vomiting	1	1.82
Rash	1	1.82

AFI = acute febrile illness; LCD = laboratory confirmed dengue; *n* = number of AFI episodes presenting with a sign or symptom of grade 3 intensity in a given category; % = *n*/55 × 100.

### Signs and symptoms of AFI because of LCD, by age group.

During the 7-day follow-up period after the onset of an AFI because of LCD, signs and symptoms of interest were almost identical from Day 1 through Day 7 in all age groups. The only variation was that one adult who had a headache/irritability on Day 1 to Day 5 did not report this symptom on Day 6 and Day 7 (Table 5).

**Table 5:** Signs and symptoms of any intensity with AFI because of LCD during the 7-day period from the onset of fever by age group

	< 5 years of age			5--11 years of age			12--17 years of age			≥ 18 years of age				
	Day 1–7			Day 1–7			Day 1–7			Day 1–5			Day 6–7	
Symptoms	*N*	*n*	%	*N*	*n*	%	*N*	*n*	%	*N*	*n*	%	*n*	%
Headache/irritability	11	9	81.82	19	17	89.47	7	7	100.00	18	18	100.00	17	94.44
Eye pain	11	7	63.64	19	12	63.16	7	5	71.43	18	10	55.56	10	55.56
Myalgia (muscle pain)	11	10	90.91	19	17	89.47	7	5	71.43	18	15	83.33	15	83.33
Arthralgia (joint pain)	11	6	54.55	19	14	73.68	7	6	85.71	18	16	88.89	16	88.89
Abdominal pain	11	7	63.64	19	8	42.11	7	5	71.43	18	11	61.11	11	61.11
Nausea	11	6	54.55	19	11	57.89	7	4	57.14	18	8	44.44	8	44.44
Vomiting	11	8	72.73	19	11	57.89	7	4	57.14	18	6	33.33	6	33.33
Rash	11	2	18.18	19	3	15.79	7	0	00.00	18	0	00.00	0	00.00
Any bleeding (skin, mouth, anus)	11	1	9.09	19	1	5.26	7	0	00.00	18	2	11.11	2	11.11
Loss of appetite	11	9	81.82	19	16	84.21	7	6	85.71	18	12	66.67	12	66.67
Fatigue/decrease in everyday activity	11	10	90.91	19	17	89.47	7	6	85.71	18	15	83.33	15	83.33
Reduced fluid intake	11	8	72.73	19	14	73.68	7	5	71.43	18	6	33.33	6	33.33

AFI = acute febrile illness; LCD = laboratory confirmed dengue; *N* = number of AFI episodes during the follow-up period; *n* = number of AFI episodes presenting with a sign or symptom of any intensity in a given category; % = *n*/*N* x 100.

There were no clinically important differences in the reporting profile of signs and symptoms across age groups. Similar to the signs and symptoms reported in the total cohort, headache/irritability, myalgia, and fatigue or decrease in everyday activity, were the most frequently reported signs and symptoms in each of the age groups, as they each occurred in more than 70.0% of AFI because of LCD. In all age groups, except those < 5 years of age, arthralgia also occurred in more than 70.0% of episodes. In addition, in all groups < 18 years of age, loss of appetite and reduced fluid intake occurred in more than 70.0% of the episodes. Vomiting was only common in the < 5 years of age group. Rash and bleeding were least reported in all age groups. A rash was observed in 5 (16.7%) episodes in children < 12 years of age and in none of the 25 episodes in teenagers and adults. Bleeding was observed in 2 (6.7%) of the episodes in children < 12 years, in none of the teenagers, and in 2 (11.1%) of the adults (Table 5).

### Signs and symptoms of grade 3 intensity of AFI because of LCD, by age group.

The incidence of symptoms with grade 3 intensity by age group did not change throughout the 7-day follow-up (Supplemental Table 3). Among the 11 episodes of AFI in the < 5 years of age group, one case was reported, each of grade 3, for headache/irritability, rash, loss of appetite, and reduced fluid intake. Among the 19 episodes in the 5–11 years of age group, four to five cases were reported, each of grade 3, for fatigue, headache/irritability, myalgia, and loss of appetite. Among the seven episodes in the 12–17 years of age group, one case was reported, each of grade 3, for headache/irritability, arthralgia, loss of appetite, and fatigue/decrease in everyday activity. Among the 18 episodes in the ≥ 18 years of age, eight cases of grade 3 fatigue were reported, six cases for myalgia, six cases for arthralgia, and five cases for headache/irritability (Supplemental Table 3).

### Signs and symptoms of AFI because of non-LCD.

Of the 183 AFI episodes because of non-LCD, the most commonly reported signs and symptoms of interest (occurring in at least 70.0% of cases) during the 7-day follow-up period included, in order of frequency: headache/irritability (*n* = 159), fatigue/decrease in everyday activity (*n* = 152), loss of appetite (*n* = 140), and myalgia (*n* = 133). As with AFI episodes because of LCD, the frequency of signs and symptoms reported in AFI episodes because of non-LCD were similar from Day 1 through Day 7 (Supplemental Table 4).

The signs and symptoms did not show any clinically important differences among age groups. Among the 54 episodes of AFI because of non-LCD in the < 5 years of age group, > 70.0% reported headache/irritability, loss of appetite, and fatigue/decrease in everyday activity. Among the 71 episodes in the 5–11 years of age group and the 34 episodes in the ≥ 18 years of age group, > 70.0% reported headache/irritability (*n* = 64; *n* = 32, respectively), myalgia (*n* = 51; *n* = 31, respectively), loss of appetite (*n* = 56; *n* = 24, respectively), and fatigue/decrease in everyday activity (*n* = 60; *n* = 28, respectively). In the ≥ 18 years of age also arthralgia was reported in > 70.0% (*n* = 31). Among the 24 episodes in the 12–17 years of age group, > 70.0% reported headache/irritability (*n* = 20), myalgia (*n* = 17), and fatigue/decrease in everyday activity (*n* = 17). Rash and any bleeding were least reported in all age groups (Supplemental Table 5).

### Signs and symptoms of grade 3 intensity of AFI because of non-LCD.

Symptoms with grade 3 intensity occurred in less than 10.0% of the 183 AFI episodes because of non-LCD. Most frequently reported were fatigue/decrease in everyday activity (*n* = 17) and headache/irritability (*n* = 16) (Supplemental Table 6).

The incidence of grade 3 intensity signs and symptoms by age group did not change throughout the 7-day follow-up. Among the 54 episodes of AFI because of non-LCD in the < 5 years of age group, the most frequently reported symptoms were loss of appetite (*n* = 5), headache/irritability (*n* = 4), fatigue/decrease in everyday activity (*n* = 4), and reduced fluid intake (*n* = 4) at about 7.0–9.0% each. Among the 71 episodes in the 5–11 years of age group, the most frequently reported symptoms were fatigue/decrease in everyday activity (*n* = 8), headache/irritability (*n* = 7), myalgia (*n* = 5), and loss of appetite (*n* = 5) at about 7.0–11.0% each. Among the 24 episodes in the 12–17 years of age group, the most frequently reported symptoms were myalgia (*n* = 2), eye pain (*n* = 1), abdominal pain (*n* = 1), and nausea (*n* = 1) at about 4.0–8.0% each. Among the 34 episodes in the ≥ 18 years of age, the most frequently reported symptoms were headache/irritability (*n* = 5), arthralgia (*n* = 7), myalgia (*n* = 6), and fatigue (*n* = 5) at about 14.0--20.0% each (Supplemental Table S7).

### Signs and symptoms associated with AFI because of LCD.

Of the signs and symptoms in the 251 AFI episodes, eye pain (*P* < 0.01), arthralgia (*P* < 0.05), and vomiting (*P* < 0.05) were found to be associated with AFI because of LCD in a univariate logistic regression, whereas only eye pain was associated in a multivariate logistic regression (*P* < 0.01) (Supplemental Table 8).

### Hospitalizations.

Of the 55 episodes of dengue, eight (14.5%) episodes resulted in hospitalization, two (3.6%) episodes in the 5–11 years of age group, 2 (3.6%) episodes in the 12–17 years of age group, and 4 (7.3%) episodes in the adult group. Of the 183 episodes of AFI because of non-LCD, seven (3.8%) episodes resulted in hospitalization, three (1.6%) episodes in the < 5 years of age group, three (1.6%) episodes in the 5–11 years of age group, and one (0.5%) episode in the adult group (Supplemental Table 9).

## DISCUSSION

This prospective 2-year cohort study conducted in the Colombo district of Sri Lanka detected an incidence of LCD of 14.2 per 1,000 person-years in the total study population, consisting of adults and children. Incidence varied between age groups from 9.2 per 1,000 person-years in adults ≥ 18 years of age to 22.7 per 1,000 person-years in children 5–11 years of age. The vast majority of the episodes (*n* = 46) were because of DENV-2, indicating this study was conducted during a DENV-2 dengue epidemic.

The dengue incidence rate we found (14.2 per 1,000 person-years) was comparable to the reported rate of suspected dengue in 2017 for the Colombo district, which was 1,419 per 100,000 population.^9^ In 2017, one of the worst outbreaks of dengue occurred in Sri Lanka; in all 25 districts, dengue incidence was significantly higher than in the previous 5 years (2012–2016). In the Colombo district, where our current study was performed, the incidence was 2.8-fold higher in 2017 than in the previous 5 years.^9^ Also, according to the International Federation of Red Cross and Red Crescent Societies, the outbreak of 2017 was the largest outbreak in three decades in Sri Lanka, with a total of 186,101 suspected dengue cases reported.^10^

Few reports of prospective studies of dengue incidence using active surveillance or even seroprevalence studies are available in Sri Lanka, especially for adults. When comparing seroprevalence data in children < 16 years of age between 2003 and 2014, it was found that seroprevalence had significantly increased (from 34.1% to 50.7%), and that the annual conversion rate was 1.5% in 2003, while it was 3.8% in 2014.^11^ In another seroprevalence study, in about 800 children < 12 years of age in an urban area in Sri Lanka from November 2008 to January 2009, more than half were already seropositive at enrollment, and the risk of primary infection was 14.0% per year.^12^ In a subsequent follow-up from November 2008 to January 2010, the incidence of clinically apparent LCD was 33.8 per 1,000 person-years (95% CI: 22.4–48.8).^13^ Although their diagnosis method of LCD was not the same as in our study, their incidence of clinically apparent LCD was somewhat higher but comparable to the incidence we observed in children < 12 years of age, which was 22.1 per 1,000 person-years on average. In our study, LCD was based on a positive RT-PCR test, while in the 2008–2010 study, LCD was based on a positive result in two of three diagnostic assays (RT-PCR, increasing levels of IgM or IgG in paired acute phase, and convalescent-phase blood samples), which likely increased their number of positive results.^13^ The total dengue incidence in the 2008–2010 study was 83.9 per 1,000 person-years; as for this incidence, in addition to clinically apparent LCD cases, children who had clinically inapparent dengue and seroconverted during the year were counted.

In Sri Lanka, dengue is endemic, and from 1991--2008 dengue epidemics occurred every few years on the background of the endemic transmission.^14^ Since then, dengue cases steadily increased according to data from the passive national surveillance system.^15^ Dominant serotypes varied throughout the years, as DENV-3 was the dominant strain from 1981--1998,^16^ both DENV-2 and DENV-3 were common in 2003–2006,^17^ and DENV-1 was the dominant serotype from 2006--2016.^18^^–^^20^ The large majority (83.6%) of LCD episodes in our study was because of DENV-2, suggesting a DENV-2 epidemic. This observation was recently confirmed in a virologic study, which showed that in 2017, during the largest dengue epidemic in Sri Lanka so far, DENV-2 was at 89.0% the dominant serotype.^9^

The clinical challenges of distinguishing dengue-related AFI from AFI because of other causes are well documented.^18^ Even the improved guidelines for the diagnosis of dengue by the WHO based on clinical criteria^1^ were found to have low specificity (65.0%) to accurately diagnose dengue when analyzed in a prospective study of patients hospitalized with fever in Sri Lanka during a DENV-1 epidemic.^18^ We also found that the signs and symptoms of interest were indeed quite similar between LCD and non-LCD cases, as both groups reported headache, fatigue, myalgia, and loss of appetite in more than 70.0% of cases. Only arthralgia was a symptom of interest for LCD but not for non-LCD, although in the non-LCD group it was also present in 64.5% (*n* = 118). The most common symptoms of LCD with grade 3 intensity were fatigue (25.5%), headache/irritability (20.0%), myalgia (18.2%), arthralgia (14.6%), and loss of appetite (14.6%). The most common symptoms with grade 3 intensity because of non-LCD were fatigue (9.3%) and headache/irritability (8.7%). In our cohort, eye pain, arthralgia, and vomiting were associated with AFI because of LCD in a univariate regression analysis, although only eye pain was also associated in a multivariate regression analysis. Interestingly, although previously eye pain has been found to be a common clinical feature of symptomatic dengue,^21^ it is not in the current WHO guidelines for the diagnosis of dengue.^1^ The explanation for this may be that eye pain is more common in younger adult (18–35 years of age) than in older adult (≥ 36 years of age) dengue patients,^22^ while the WHO guidelines do not differentiate by age.^1^ Overall, it is not possible to distinguish between LCD and non-LCD based on clinical presentation alone. This emphasizes the need for laboratory diagnosis to distinguish between the causes of AFI, as knowing the cause of the AFI allows for appropriate treatment at the individual level and targeted prevention at the population level.

One study limitation was that our dengue definition was based on ≥ 2 days of AFI regardless of other dengue symptoms. This definition is problematic as few participants would undergo ≥ 2 days of fever without self-administering antipyretics, and parents would very likely administer them to their children. In addition, we know from a large study assessing dengue presentation that, although only a low percentage of dengue patients present without fever (3.4% of 14,756 patients), the large majority of those did have hemorrhagic manifestations (65.7%), and a substantial number of them had failure of at least one organ (30.7%).^23^ Also, as discussed earlier, a substantial number of clinically inapparent cases will be missed. Another limitation of the study is the missed opportunity to identify the cause of the AFI in non-LCD cases. It would have made the study more informative to both health authorities and researchers and clinicians interested in AFI in Sri Lanka in general.

**Figure 2.**
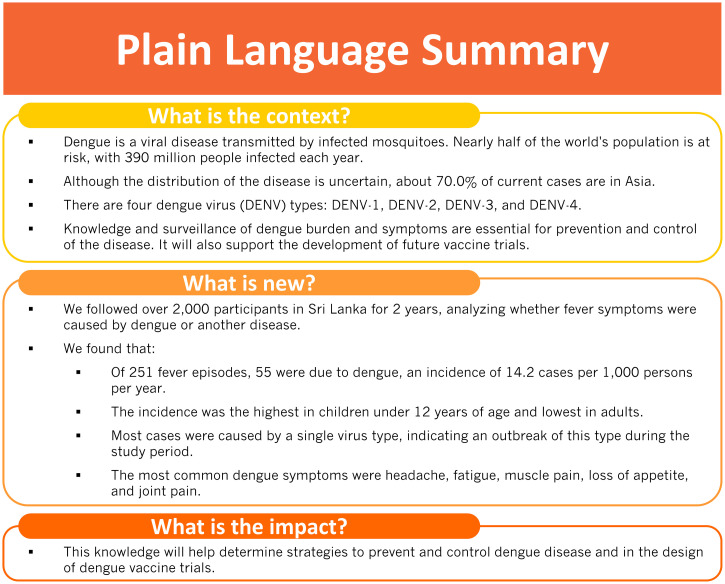
Plain language summary. This figure appears in color at www.ajtmh.org.

In conclusion, this study occurred during a dengue epidemic because of DENV-2. A total of 55 LCD episodes were found, mostly in children ≤ 11 years, and incidence was 14.2 per 1,000 person-years, ranging from 9.2 to 22.7 per 1,000 person-years depending on age. Our study contributes to the current knowledge of the epidemiology of dengue infection in Sri Lanka. This knowledge will help inform surveillance strategies as well as the design of dengue vaccine efficacy trials in Southeast Asia. The findings of this study are summarized in plain language in Figure 2.

## Supplemental tables


Supplemental materials

## References

[1] World Health Organization , 2009. *Dengue Guidelines for Diagnosis, Treatment, Prevention and Control: New Edition*. Report No.: WHO/HTM/NTD/DEN/2009.1. Geneva, Switzerland: WHO. Available at: https://apps.who.int/iris/bitstream/handle/10665/44188/9789241547871_eng.pdf. Accessed February 2, 2021. 23762963

[2] Stanaway JD , 2016. The global burden of dengue: an analysis from the Global Burden of Disease Study 2013. Lancet Infect Dis 16: 712–723. 26874619 10.1016/S1473-3099(16)00026-8 PMC5012511

[3] World Health Organization , 2020. *Dengue and Severe Dengue*. Geneva, Switzerland: WHO. Available at: https://www.who.int/news-room/fact-sheets/detail/dengue-and-severe-dengue. Accessed February 2, 2021.

[4] World Health Organization , 2020. *Global Strategy for Dengue Prevention and Control. 2012–2020*. Geneva, Switzerland: WHO. Available at: https://apps.who.int/iris/bitstream/handle/10665/75303/9789241504034_eng.pdf. Accessed February 2, 2021.

[5] Brady OJ Gething PW Bhatt S Messina JP Brownstein JS Hoen AG Moyes CL Farlow AW Scott TW Hay SI, 2012. Refining the global spatial limits of dengue virus transmission by evidence-based consensus. PLoS Negl Trop Dis 6: e1760. 22880140 10.1371/journal.pntd.0001760 PMC3413714

[6] Bhatt S , 2013. The global distribution and burden of dengue. Nature 496: 504–507. 23563266 10.1038/nature12060 PMC3651993

[7] International Council for Harmonization , 2016. *ICH Harmonised Guideline Integrated Addendum to ICH E6(R1): Guideline for Good Clinical Practice ICH E6(R2) ICH Consensus Guideline*. Geneva, Switzerland: ICH. Available at: https://database.ich.org/sites/default/files/E6_R2_Addendum.pdf. Accessed April 26, 2021.

[8] U.S. National Library of Medicine , 2015. *A Cohort Study to Determine the Incidence of Dengue Fever and to Build Capacity for Dengue Vaccine Trials in Dengue-endemic Regions of South Asia*. Available at: https://clinicaltrials.gov/ct2/show/NCT02570152. Accessed February 9, 2021.

[9] Tissera HA , 2020. Severe dengue epidemic, Sri Lanka, 2017. Emerg Infect Dis 26: 682–691. 32186490 10.3201/eid2604.190435 PMC7101108

[10] International Federation of Red Cross and Red Crescent Societies (IFRC) , 2018. *Sri Lanka/Dengue DREF Final Report (MDRLK007)*. Report No.: MDRLK007. Geneva, Switzerland: IFRC. Available at: https://reliefweb.int/report/sri-lanka/sri-lanka-dengue-dref-final-report-mdrlk007. Accessed February 9, 2021.

[11] Jeewandara C , 2015. Change in dengue and Japanese encephalitis seroprevalence rates in Sri Lanka. PLoS One 10: e0144799. 26696417 10.1371/journal.pone.0144799 PMC4687926

[12] Tam CC Tissera H de Silva AM de Silva AD Margolis HS Amarasinge A, 2013. Estimates of dengue force of infection in children in Colombo, Sri Lanka. PLoS Negl Trop Dis 7: e2259. 23755315 10.1371/journal.pntd.0002259 PMC3674987

[13] Tissera H Amarasinghe A de Silva AD Kariyawasam P Corbett KS Katzelnick L Tam C Letson GW Margolis HS de Silva AM, 2014. Burden of dengue infection and disease in a pediatric cohort in urban Sri Lanka. Am J Trop Med Hyg 91: 132–137. 24865684 10.4269/ajtmh.13-0540 PMC4080552

[14] Kulatilaka TA Jayakuru WS, 1998. Control of dengue/dengue haemorrhagic fever in Sri Lanka. Dengue Bull 22: 53–61.

[15] Ministry of Health - Epidemiology Unit - Sri Lanka , 2019. *Distribution of Notification (H399). Dengue Cases by Month*. Colombo, Sri Lanka: Ministry of Health. Available at: https://www.epid.gov.lk/web/index.php?option=com_casesanddeaths&Itemid=448&lang=en#. Accessed February 9, 2021.

[16] Messer WB Gubler DJ Harris E Sivananthan K de Silva AM, 2003. Emergence and global spread of a dengue serotype 3, subtype III virus. Emerg Infect Dis 9: 800–809. 12899133 10.3201/eid0907.030038 PMC3023445

[17] Kanakaratne N Wahala WM Messer WB Tissera HA Shahani A Abeysinghe N de Silva AM Gunasekera M, 2009. Severe dengue epidemics in Sri Lanka, 2003–2006. Emerg Infect Dis 15: 192–199. 19193262 10.3201/eid1502.080926 PMC2662655

[18] Bodinayake CK , 2018. Evaluation of the WHO 2009 classification for diagnosis of acute dengue in a large cohort of adults and children in Sri Lanka during a dengue-1 epidemic. PLoS Negl Trop Dis 12: e0006258. 29425194 10.1371/journal.pntd.0006258 PMC5823472

[19] Raut R , 2019. Dengue type 1 viruses circulating in humans are highly infectious and poorly neutralized by human antibodies. Proc Natl Acad Sci USA 116: 227–232. 30518559 10.1073/pnas.1812055115 PMC6320508

[20] Tissera HA , 2011. New dengue virus type 1 genotype in Colombo, Sri Lanka. Emerg Infect Dis 17: 2053–2055. 22099096 10.3201/eid1711.101893 PMC3310553

[21] Tomashek KM , 2017. Clinical and epidemiologic characteristics of dengue and other etiologic agents among patients with acute febrile illness, Puerto Rico, 2012–2015. PLoS Negl Trop Dis 11: e0005859. 28902845 10.1371/journal.pntd.0005859 PMC5597097

[22] Low JGH , 2011. The early clinical features of dengue in adults: challenges for early clinical diagnosis. PLoS Negl Trop Dis 5: e1191. 21655307 10.1371/journal.pntd.0001191 PMC3104968

[23] Tukasan C Furlan NB Estofolete CF Nogueira ML da Silva NS, 2017. Evaluation of the importance of fever with respect to dengue prognosis according to the 2009 WHO classification: a retrospective study. BMC Infect Dis 17: 6. 28052760 10.1186/s12879-016-2128-4 PMC5209937

