# Supplementary material for: The Burden of Acute Febrile Illness Attributable to Dengue Virus Infection in Sri Lanka: A Single-Center 2-Year Prospective Cohort Study (2016–2019)
Source: Am J Trop Med Hyg. 2021 Nov 1;106(1):160–7. doi: 10.4269/ajtmh.21-0604 (PMC8733532; doi:10.4269/ajtmh.21-0604)
Supplement: Supplementary file 1 [file tpmd210604.SD1.pdf]

Supplementary material

Table S1 Grading of symptoms

Intensity scales for solicited symptoms (excluding bleeding and reduced fluid intake):

Grade	Symptom
0 (none)	No discomfort
1 (mild)	Any solicited symptom, which is easily tolerated by the participant, causing minimal discomfort, and not interfering with everyday activities
2 (moderate)	Any solicited symptom, which is sufficiently discomforting to interfere with normal everyday activities
3 (severe)	Any solicited symptom, which prevents normal, everyday activities. In a young child, such a solicited symptom would, for example, prevent attendance at school/kindergarten/daycare center and would cause the parent(s)/legally acceptable representative(s) to seek medical advice. In adults/adolescents, such a solicited symptom would, for example, prevent attendance at work/school and would necessitate the administration of corrective therapy

Intensity scales for bleeding:

Grade	Symptom
0 (none)	No bleeding or bruising
1 (mild)	Small skin bleed or easy bruising
2 (moderate)	Nose bleed or gum bleed not requiring medical attention
3 (severe)	Bleeding that requires medical attention

Intensity scales for reduced fluid intake:

Grade	Symptom*
0 (none)	Normal feeding
1 (mild)	Drinks a little less than usual
2 (moderate)	Does not drink well
3 (severe)	Lethargic, does not drink

*Wording intended for infants.

Table S2 Percentage and incidence rates of AFI due to LCD per DENV serotype

**Serotype**	**n**	**%**	**Incidence rate per 1,000 person-years (95% CI: LL–UL)**
DENV-1	6	10.9	1.55 (0.57– 3.38)
DENV-2	46	83.6	11.89 (8.71–15.86)
DENV-3	3	5.5	0.78 (0.16–2.27)
DENV-4	0	0.0	0.00 (0.00–0.95)

AFI, Acute Febrile illness; DENV, dengue virus; LCD, Laboratory Confirmed Dengue; LL, lower limit; N, number of AFI episodes due to LCD during the follow-up period (N= 55); n, number of first events reported during the follow-up period at risk; UL, upper limit; 95% CI, 95% confidence interval; %, n/N x 100.

Table S3 Signs or symptoms of grade 3 intensity with AFI due to LCD during the 7-day period from the onset of fever by age group

	< 5 years of age			5 to 11 years of age			12 to 17 years of age			≥ 18 years of age		
	Day 1 to 7			Day 1 to 7			Day 1 to 7			Day 1 to 7		
**Symptoms**	**N**	**n**	**%**	**N**	**n**	**%**	**N**	**n**	**%**	**N**	**n**	**%**
Headache / irritability	11	1	9.09	19	4	21.05	7	1	14.29	18	5	27.78
Eye pain	11	0	0.00	19	2	10.53	7	0	0.00	18	0	0.00
Myalgia (muscle pain)	11	0	0.00	19	4	21.05	7	0	0.00	18	6	33.33
Arthralgia (joint pain)	11	0	0.00	19	1	5.26	7	1	14.29	18	6	33.33
Abdominal pain	11	0	0.00	19	1	5.26	7	0	0.00	18	1	5.56
Vomiting	11	0	0.00	19	1	5.26	7	0	0.00	18	0	0.00
Rash	11	1	9.09	19	0	0.00	7	0	0.00	18	0	0.00
Loss of appetite	11	1	9.09	19	4	21.05	7	1	14.29	18	2	11.11
Fatigue/decrease in everyday activity	11	0	0.00	19	5	26.32	7	1	14.29	18	8	44.44
Reduced fluid intake	11	1	9.09	19	1	5.26	7	0	0.00	18	2	11.11

AFI, Acute Febrile Illness; LCD, Laboratory Confirmed Dengue; N, number of participants at risk during the follow-up period; n, number of AFI episodes presenting with a sign or symptom of grade 3 intensity in a given category; %, n/N x 100.

Table S4 Signs or symptoms of any intensity with AFI due to non-LCD during the 7-day period from the onset of fever

		Day 1 to 3		Day 4		Day 5 to 6		Day 7	
**Symptoms**	**N**	**n**	**%**	**n**	**%**	**n**	**%**	**n**	**%**
Headache / irritability	183	159	86.89	157	85.79	158	86.34	158	86.34
Fatigue/decrease in everyday activity	183	152	83.06	150	81.97	151	82.51	150	81.97
Loss of appetite	183	140	76.50	138	75.41	139	75.96	137	74.86
Myalgia (muscle pain)	183	133	72.68	131	71.58	132	72.13	132	72.13
Arthralgia (joint pain)	183	118	64.48	116	63.39	117	63.93	117	63.93
Reduced fluid intake	183	105	57.38	103	56.28	103	56.28	102	55.74
Nausea	183	92	50.27	90	49.18	91	49.73	91	49.73
Abdominal pain	183	87	47.54	86	46.99	87	47.54	87	47.54
Eye pain	183	80	43.72	78	42.62	79	43.17	78	42.62
Vomiting	183	71	38.80	70	38.25	71	38.80	71	38.80
Rash	183	7	3.83	7	3.83	7	3.83	7	3.83
Any bleeding (skin, mouth, anus)	183	6	3.28	6	3.28	6	3.28	6	3.28

AFI, Acute Febrile Illness; LCD, Laboratory Confirmed Dengue; N, number of AFI episodes during the follow-up period; n, number of AFI episodes presenting with a sign or symptom of any intensity in a given category; %, n/N x 100.

Table S5 Signs and symptoms of any intensity with AFI due to non-LCD during the 7-day period from the onset of fever by age group

			Day 1 to 3		Day 4		Day 5 to 6		Day 7	
**Age group**	**Symptoms**	**N**	**n**	**%**	**n**	**%**	**n**	**%**	**n**	**%**
< 5 years of age	Headache / irritability	54	42	77.78	41	75.93	42	77.78	42	77.78
	Eye pain	54	17	31.48	16	29.63	17	31.48	17	31.48
	Myalgia (muscle pain)	54	33	61.11	32	59.26	33	61.11	33	61.11
	Arthralgia (joint pain)	54	30	55.56	29	53.70	30	55.56	30	55.56
	Abdominal pain	54	28	51.85	27	50.00	28	51.85	28	51.85
	Nausea	54	28	51.85	27	50.00	28	51.85	28	51.85
	Vomiting	54	26	48.15	25	46.30	26	48.15	26	48.15
	Rash	54	2	3.70	2	3.70	2	3.70	2	3.70
	Any bleeding (skin, mouth, anus)	54	1	1.85	1	1.85	1	1.85	1	1.85
	Loss of appetite	54	44	81.48	43	79.63	44	81.48	43	79.63
	Fatigue/decrease in everyday activity	54	45	83.33	44	81.48	45	83.33	45	83.33
	Reduced fluid intake	54	29	53.70	28	51.85	29	53.70	29	53.70
5 to 11 years of age	Headache / irritability	71	64	90.14	64	90.14	64	90.14	64	90.14
	Eye pain	71	35	49.30	35	49.30	35	49.30	35	49.30
	Myalgia (muscle pain)	71	51	71.83	51	71.83	51	71.83	51	71.83
	Arthralgia (joint pain)	71	44	61.97	44	61.97	44	61.97	44	61.97
	Abdominal pain	71	41	57.75	41	57.75	41	57.75	41	57.75
	Nausea	71	38	53.52	38	53.52	38	53.52	38	53.52

Age group	Symptoms		Day 1 to 3		Day 4		Day 5 to 6		Day 7	
		N	n	%	n	%	n	%	n	%
	Vomiting	71	29	40.85	29	40.85	29	40.85	29	40.85
	Rash	71	3	4.23	3	4.23	3	4.23	3	4.23
	Any bleeding (skin, mouth, anus)	71	4	5.63	4	5.63	4	5.63	4	5.63
	Loss of appetite	71	56	78.87	56	78.87	56	78.87	56	78.87
	Fatigue/decrease in everyday activity	71	60	84.51	60	84.51	60	84.51	60	84.51
	Reduced fluid intake	71	43	60.56	43	60.56	43	60.56	43	60.56
12 to 17 years of age	Headache / irritability	24	21	87.50	20	83.33	20	83.33	20	83.33
	Eye pain	24	13	54.17	12	50.00	12	50.00	11	45.83
	Myalgia (muscle pain)	24	18	75.00	17	70.83	17	70.83	17	70.83
	Arthralgia (joint pain)	24	13	54.17	12	50.00	12	50.00	12	50.00
	Abdominal pain	24	9	37.50	9	37.50	9	37.50	9	37.50
	Nausea	24	13	54.17	12	50.00	12	50.00	12	50.00
	Vomiting	24	6	25.00	6	25.00	6	25.00	6	25.00
	Rash	24	2	8.33	2	8.33	2	8.33	2	8.33
	Any bleeding (skin, mouth, anus)	24	1	4.17	1	4.17	1	4.17	1	4.17
	Loss of appetite	24	16	66.67	15	62.50	15	62.50	14	58.33
	Fatigue/decrease in everyday activity	24	19	79.17	18	75.00	18	75.00	17	70.83
	Reduced fluid intake	24	14	58.33	13	54.17	12	50.00	11	45.83
≥ 18 years of	Headache / irritability	34	32	94.12	32	94.12	32	94.12	32	94.12

Age group	Symptoms	N	Day 1 to 3		Day 4		Day 5 to 6		Day 7	
			n	%	n	%	n	%	n	%
age	Eye pain	34	15	44.12	15	44.12	15	44.12	15	44.12
	Myalgia (muscle pain)	34	31	91.18	31	91.18	31	91.18	31	91.18
	Arthralgia (joint pain)	34	31	91.18	31	91.18	31	91.18	31	91.18
	Abdominal pain	34	9	26.47	9	26.47	9	26.47	9	26.47
	Nausea	34	13	38.24	13	38.24	13	38.24	13	38.24
	Vomiting	34	10	29.41	10	29.41	10	29.41	10	29.41
	Loss of appetite	34	24	70.59	24	70.59	24	70.59	24	70.59
	Fatigue/decrease in everyday activity	34	28	82.35	28	82.35	28	82.35	28	82.35
	Reduced fluid intake	34	19	55.88	19	55.88	19	55.88	19	55.88

AFI, Acute Febrile Illness; LCD, Laboratory Confirmed Dengue; N, number of AFI episodes during the follow-up period; n, number of AFI episodes presenting with a sign or symptom of any intensity in a given category; %, n/N x 100.

Table S6 Signs or symptoms of grade 3 intensity with AFI due to non-LCD during the 7-day period from the onset of fever

		Day 1 to 3		Day 4		Day 5 to 7	
**Symptoms**	**N**	**n**	**%**	**n**	**%**	**n**	**%**
Headache/irritability	183	16	8.74	15	8.20	16	8.74
Eye pain	183	5	2.73	5	2.73	5	2.73
Myalgia (muscle pain)	183	14	7.65	13	7.10	14	7.65
Arthralgia (joint pain)	183	12	6.56	11	6.01	12	6.56
Abdominal pain	183	4	2.19	3	1.64	4	2.19
Nausea	183	4	2.19	3	1.64	4	2.19
Vomiting	183	4	2.19	3	1.64	4	2.19
Rash	183	1	0.55	1	0.55	1	0.55
Loss of appetite	183	11	6.01	11	6.01	11	6.01
Fatigue/decrease in everyday activity	183	17	9.29	16	8.74	17	9.29
Reduced fluid intake	183	5	2.73	5	2.73	5	2.73

AFI, Acute Febrile Illness; LCD, Laboratory Confirmed Dengue; N, number of ADI episodes during the follow-up period; n, number of AFI episodes presenting with a sign or symptom of grade 3 intensity in a given category; %, n/N x 100.

Table S7 Signs or symptoms of grade 3 intensity with AFI due to non-LCD during the 7-day period from the onset of fever by age group

			Day 1 to 3		Day 4		Day 5 to 7	
**Age group**	**Symptoms**	**N**	**n**	**%**	**n**	**%**	**n**	**%**
< 5 years of age	Headache / irritability	54	4	7.41	3	5.56	4	7.41
	Eye pain	54	2	3.70	2	3.70	2	3.70
	Myalgia (muscle pain)	54	1	1.85	0	0.00	1	1.85
	Arthralgia (joint pain)	54	1	1.85	0	0.00	1	1.85
	Abdominal pain	54	1	1.85	0	0.00	1	1.85
	Nausea	54	2	3.70	1	1.85	2	3.70
	Vomiting	54	1	1.85	0	0.00	1	1.85
	Rash	54	1	1.85	1	1.85	1	1.85
	Loss of appetite	54	5	9.26	5	9.26	5	9.26
	Fatigue/decrease in everyday activity	54	4	7.41	3	5.56	4	7.41
	Reduced fluid intake	54	4	7.41	4	7.41	4	7.41
5 to 11 years of age	Headache / irritability	71	7	9.86	7	9.86	7	9.86
	Eye pain	71	2	2.82	2	2.82	2	2.82
	Myalgia (muscle pain)	71	5	7.04	5	7.04	5	7.04
	Arthralgia (joint pain)	71	4	5.63	4	5.63	4	5.63
	Abdominal pain	71	2	2.82	2	2.82	2	2.82
	Nausea	71	1	1.41	1	1.41	1	1.41
	Vomiting	71	3	4.23	3	4.23	3	4.23
	Loss of appetite	71	5	7.04	5	7.04	5	7.04
	Fatigue/decrease in everyday activity	71	8	11.27	8	11.27	8	11.27
	Reduced fluid intake	71	1	1.41	1	1.41	1	1.41

Age group	Symptoms	N	Day 1 to 3		Day 4		Day 5 to 7	
			n	%	n	%	n	%
12 to 17 years of age	Eye pain	24	1	4.17	1	4.17	1	4.17
	Myalgia (muscle pain)	24	2	8.33	2	8.33	2	8.33
	Abdominal pain	24	1	4.17	1	4.17	1	4.17
	Nausea	24	1	4.17	1	4.17	1	4.17
≥ 18 years of age	Headache / irritability	34	5	14.71	5	14.71	5	14.71
	Myalgia (muscle pain)	34	6	17.65	6	17.65	6	17.65
	Arthralgia (joint pain)	34	7	20.59	7	20.59	7	20.59
	Loss of appetite	34	1	2.94	1	2.94	1	2.94
	Fatigue/decrease in everyday activity	34	5	14.71	5	14.71	5	14.71

AFI, Acute Febrile Illness; LCD, Laboratory Confirmed Dengue; N, number of AFI episodes during the follow-up period; n, number of AFI episodes presenting with a sign or symptom of grade 3 intensity in a given category; %, n/N x 100.

Table S8 Logistic regressions associate sign or symptom with AFI due to LCD

Univariate logistic regression

Sign or symptom	Estimate	P-value
Headache / irritability	1.0537	0.0558
Eye pain	0.8323	0.0079
Myalgia (muscle pain)	1.0000	0.0153
Arthralgia (joint pain)	0.7374	0.0348
Abdominal pain	0.4607	0.1341
Nausea	0.2113	0.4893
Vomiting	0.6528	0.0341
Rash	0.9933	0.1016
Any bleeding (skin, mouth, anus)	0.9097	0.1710
Loss of appetite	0.3348	0.3566
Fatigue/decrease in everyday activity	0.6561	0.1358
Reduced fluid intake	0.2418	0.4358

Multivariate logistic regression

Sign or symptom	Estimate	P-value
Eye pain	0.8323	0.0079

N = 251 observations

Table S9 Frequency and percentage of hospitalization

		Age group								Overall	
		< 5 years of age		5 – 11 years of age		12 – 17 years of age		≥ 18 years of age			
**Reason**	**N**	**n**	**%**	**n**	**%**	**n**	**%**	**n**	**%**	**n**	**%**
Dengue	55	0	0.00	2	3.64	2	3.64	4	7.27	8	14.55
Unknown viral fever	183	3	1.64	3	1.64	0	0.00	1	0.55	7	3.83

N, total number of episodes for the reason category; n, number of hospitalizations for the category; %, n/N x 100
